# Advances in the Management of Infectious Diseases

**DOI:** 10.3390/idr17020026

**Published:** 2025-03-14

**Authors:** Rabeea F. Omar, Sylvie Trottier, Sachiko Sato, Marc Ouellette, Michel G. Bergeron

**Affiliations:** Centre de Recherche en Infectiologie (CRI) de l’Université Laval, CHU de Québec-Université Laval (CHUL), Quebec City, QC G1V 4G2, Canada; rabeea.omar@crchudequebec.ulaval.ca (R.F.O.); sylvie.trottier@crchudequebec.ulaval.ca (S.T.); sachiko.sato@crchudequebec.ulaval.ca (S.S.); michel.g.bergeron@crchudequebec.ulaval.ca (M.G.B.)

The landscape of infectious diseases has dramatically evolved since the 1970s and the advent of antimicrobials, which heralded a new era in medical history. As we navigate the mid-2020s, this field continues to present daunting challenges to global health. In the last several years, we have witnessed the rise of new infections, such as HIV, SARS, H1N1, and most recently, COVID-19, which continues to impact society five years after its initial emergence in 2020 [1]. The widespread use of antimicrobial drugs has led to the emergence of resistance, now recognized as an important global health challenge [2]. This editorial reflects on the progress made over the past five decades and highlights current advancements in three fundamental pillars of infectious disease research: prevention, diagnosis, and treatment. These interconnected disciplines form the cornerstone of our efforts to combat both existing and emerging pathogens.

Prevention aims at protecting individuals and populations from pathogen transmission and illness through a range of strategies. Vaccine development has provided remarkable progress in that regard, with the successful implementation of combination vaccines like the MMR (measles, mumps, and rubella) in 1971 or the hepatitis B vaccine in 1982. Recent breakthroughs in mRNA technology, as demonstrated by COVID-19 vaccines, have also opened new avenues for rapid and flexible vaccine production [3]. The development of plant-based vaccines represents another promising approach, potentially leading to efficient and cost-effective vaccines against a diverse range of pathogens [4]. Furthermore, the establishment of numerous animal models of infection is providing a critical platform for the in-depth investigation of host–pathogen dynamics, facilitating vaccine development and testing [5]. Significant advancements are also being made in the development of multipurpose prevention technologies, such as vaginal microbicidal gel formulations designed to protect women against sexually transmitted infections [6].

Prevention begins with early detection, and coordinated global responses are critical to mitigating outbreaks before they escalate. Innovative approaches like wastewater surveillance have also emerged as powerful tools for early outbreak detection. By monitoring pathogens in wastewater, public health officials can identify infection trends even before symptoms appear in communities, enabling timely interventions [7]. This method, which gained prominence during the COVID-19 pandemic, is now being expanded to monitor other infectious threats globally [7]. These surveillance systems are complemented by vaccination programs aimed at achieving high coverage rates, such as Canada’s goal of 95% childhood vaccine coverage by 2025, aligned with the WHO’s global disease elimination targets [8]. Together, these efforts reflect a growing recognition that prevention is not only about individual immunization or hygiene practices but also building comprehensive systems to detect, monitor, and respond to emerging threats. By strengthening these frameworks, global health organizations aim to enhance resilience against future pandemics and safeguard public health on a global scale.

Finally, emerging tools such as personalized medicine—along with the optimization of two classic and highly effective approaches, medications and vaccines—are being tailored to meet the specific prevention needs of vulnerable populations. For example, the genetic marker HLA-B*5701 is used to predict allergic reactions to the antiretroviral drug abacavir [9]. Recently, new vaccines against respiratory syncytial virus (RSV) which were approved for use in two especially vulnerable populations—newborn infants, through vaccination of their pregnant mothers, and older adults [10,11]—illustrate these recent advances in infectious disease control and prevention.

The diagnosis of infectious diseases has undergone a remarkable transformation, evolving from classical culture methods to cutting-edge molecular technologies. Historically, the identification of pathogens relied on techniques such as microbial culture on agar plates, which became the cornerstone of diagnostic microbiology during the late 19th century [12]. These methods, while foundational, were time-consuming and limited in scope. Serologic testing, introduced in the early 20th century, further enhanced diagnostic capabilities by enabling the detection of antibodies or antigens associated with specific infections, such as syphilis and hepatitis B. Introduced in the late 1970s and 1980s, standardized and automated identification systems with antimicrobial sensitivity testing improved the accuracy and speed of bacterial identification, but these were still based on Pasteur-era methods of microorganism culturing and isolation [13,14]. The development of the polymerase chain reaction (PCR) in the 1980s introduced a rapid and highly sensitive technique for detecting pathogens by amplifying their genetic material [12]. During the COVID-19 pandemic, PCR-based diagnostics were rapidly adopted on a global scale, enabling widespread testing and timely identification of SARS-CoV-2 infections for rapid intervention. This demonstrated the power of molecular diagnostics in responding to emerging infectious threats. Looking ahead, Next-Generation Sequencing (NGS) is poised to further redefine pathogen detection. NGS offers an unbiased and comprehensive approach by enabling the sequencing of all genetic material in a sample, allowing for the simultaneous identification of bacteria, fungi, viruses, and parasites without prior assumptions about the causative agent [15]. This technology holds immense promise for diagnosing rare or atypical infections and characterizing antimicrobial resistance genes. As the cost continues to decline and bioinformatic tools improve, NGS is expected to become a routine diagnostic tool, offering unparalleled precision and speed in identifying infectious agents.

Treatment approaches in infectious diseases continue to evolve, driven by the constant emergence of new pathogens and their genetic evolution, which leads to increased transmissibility and development of antimicrobial resistance [16]. Furthermore, antimicrobials can be limited in effectiveness if deemed too toxic or not tolerated due to side effects. Other factors, such as the route of administration and frequency of intake, also impact adherence and, consequently, efficacy. In this regard, the history of HIV treatment is particularly illustrative. AZT, the first antiretroviral approved to treat HIV, required dosing up to five times daily, caused bone marrow toxicity, induced metabolic complications, was poorly tolerated, and rapidly lost its efficacy in monotherapy due to the development of resistance [17]. Its use in combination with other antiretroviral drugs decreased the dosing frequency, toxicity, side effects, and risk of resistance. It became a basic component of the first highly active antiretroviral therapies (HAARTs) established in 1996 to control HIV infection [18]. Sustained efforts have been made to develop drugs suitable for the long-term treatment of this chronic infection. Today, injectable treatment with a combination of two long-acting antiretroviral drugs is available for administration every two months, offering excellent efficacy, toxicity, and tolerance profiles [19]. Focusing on HIV viral reservoirs also brings hope for virus eradication and significant progress towards a cure [20].

Progress is also being made against other infectious diseases. For example, researchers are exploring compounds from unexpected sources, like the Canadian boreal forest, which shows promising antimalarial activity [21]. The use of bacteriophages as an alternative to traditional antibiotics is also gaining momentum, especially for treating multidrug-resistant infections [22]. Advances in genomic sequencing and bioinformatics are enabling a deeper understanding of the mechanisms driving antimicrobial resistance, allowing for more targeted drug development [23]. Furthermore, the concept of antibiotic stewardship is being emphasized to preserve the efficacy of existing treatments [24]. For emerging threats such as mpox, efforts are underway to improve diagnostics and develop new medical countermeasures [25]. As we confront the ongoing challenge of emerging and evolving pathogens, the field of infectious diseases remains dynamic. Researchers are continuously adapting strategies to stay ahead of microbial threats.

In 2025, infectious diseases remain a leading cause of death globally. Each microbe has its unique ecological niche, own transmission mode, and range of clinical manifestations, making prevention, diagnosis, and treatment challenging. Artificial Intelligence (AI) could help manage this complexity by analyzing large datasets, such as epidemiological data, to predict outbreaks and enable timely interventions. Machine learning may also identify characteristics missed by humans. Moreover, AI could work alongside gene sequencing to identify pathogens and mutations linked to antimicrobial resistance and could aid in discovering new antimicrobial candidates, thus facilitating the development of more effective treatments [26].

Finally, over the past five decades, the training of graduate and medical students, postdoctoral fellows, physicians, and highly qualified personnel has been pivotal to the evolution of infectious disease research and development. These highly trained individuals have contributed to shaping global health strategies and research agendas by occuping positions of leadership in academia, industry, government, and non-governmental organizations. As we look forward, we are optimistic about the continued impact of these trained professionals in addressing future global health challenges. To the research community and all those who have contributed to tackling infectious diseases: your collective efforts have been instrumental in advancing our understanding and control of these complex health threats and improving the health and quality of life of humankind. As we begin this new decade of research, we anticipate further innovations in the prevention, diagnosis, and treament of infectious disease, building on the strong foundation laid during the last 50 years.
